# Efficient extraction of cucurbitacins from *Diplocyclos palmatus* (L.) C. Jeffrey: Optimization using response surface methodology, extraction methods and study of some important bioactivities

**DOI:** 10.1038/s41598-020-58924-5

**Published:** 2020-02-07

**Authors:** S. B. Patel, U. A. Attar, D. M. Sakate, S. G. Ghane

**Affiliations:** 1 Plant Physiology Laboratory, Department of Botany, Shivaji University, Kolhapur, 416 004 Maharashtra India; 2 Department of Statistics, Shivaji University, Kolhapur, 416 004 Maharashtra India

**Keywords:** Biochemistry, Plant sciences

## Abstract

*Diplocyclos palmatus* (L.) C. Jeffrey is an important medicinal plant used in several reproductive medicines. It serves as a wide source of tetracyclic triterpens called cucurbitacins. Response surface methodology (RSM) with Box-Behnken design (BBD) was studied to optimize the production of cucurbitacins. RSM put forth the ideal conditions such as 1:30 SS ratio (g/mL), 80 rpm (mixing extraction speed), 150 µm mean particle size, 30 min extraction time and 50 ^°^C using chloroform in continuous shaking extraction (CSE) and showed the highest cucurbitacin I (CUI) content (2.345 ± 0.1686 mg/g DW). Similarly, the highest yield of cucurbitacin B (CUB) (1.584 ± 0.15 mg/g DW) was recorded at ideal conditions (1:40 g/mL SS ratio and 60 min time and others similar to CUI). Among the tested extraction methods, the highest CUI, CUB, and CUI + B yield (1.437 ± 0.03, 0.782 ± 0.10, 2.17 ± 0.35 mg/g DW, respectively) as well as promising DPPH radical scavenging activity (25.06 ± 0.1 µgAAE/g DW) were recorded from the SBAE (steam bath assisted extraction). In addition, MAE and UAE revealed the highest inhibition of α-amylase (68.68%) and α-glucosidase (56.27%) enzymes, respectively. Fruit extracts showed potent anticancer activity against breast (MCF-7) and colon (HT-29) cancer cell lines (LC_50_ – 44.27 and 46.88 µg/mL, respectively). Our study proved that SS ratio, particle size and temperature were the most positively influencing variables and served to be the most efficient for the highest recovery of CUI and CUB. Based on the present study, the fruits of *D. palmatus* were revealed as a potent antioxidant, anti-diabetic and anticancer bio-resource that could be explored further to develop novel drug to manage diabetes, cancer and oxidative stress related disorders.

## Introduction

The plants from the family *Cucurbitaceae* are commonly called as melons, squashes, and gourds which are traditionally used in the human diet. It comprises 122 genera and 940 species of which 31 genera and 94 species are found in India^1^. *Diplocyclos palmatus* (L.) C. Jeffrey is a slender-stemmed tendril climber commonly called as Shivlingi. Traditionally, this plant has been used in the folk medicine and possesses several activities such as gynaecological, anti-asthmatic, anti-convulsant, anti-venom, anti-inflammatory, androgenic and antioxidant^2–4^. The family *Cucurbitaceae* has been recognized as a rich source of bitter compounds, called cucurbitacins^5^. The cucurbitacins are highly unsaturated triterpenes containing many keto-, hydroxy- and acetoxy-groups. They are the major active components in traditional medicine, herbal remedies and *Pharmacopoeia* to treat various health issues such as hepatoprotective, anti-fertility, diabroticites cardiovascular and anti-inflammatory activities^6^. In a recent study, cucurbitacin I (CUI) has been identified as a strong inhibitor of the JAK2/STAT3 signaling pathway (a common oncogenic signaling pathway), which is constitutively activated in many types of cancer; hence, it is considered as a milestone in cancer therapy^7^. Similarly, cucurbitacin B (CUB) also exhibits strong inhibitory activity against several breast (MDA-MB-231, ZR-75–1, BT474), hepatic carcinoma (BEL-7402), pancreatic (MiaPaCa-2, PL45), and colon (SW480) cancer cell lines. In addition, CUB exhibits potent pharmacological activities such as antitumor, anti-hepatic and immune-potentiating effects^8,9^. Cancer is considered as life threatening disease across the world^10^. Hence, a high demand of the drugs for new therapies to treat or prevent this life threatening disease is the need of the time^11^. Natural drugs are more preferred in the cancer treatment as chemo- and radiation therapy have several ill effects on human beings^12^. Plant based metabolites have been studied extensively for their anticancer properties^13^. *Cucurbitaceae* is a vital source of cucurbitacins showed anti-proliferative potential on human cancer cell lines and tumor xenografts, including breast, prostate, lung, uterine cervix, liver, skin and brain cancers^14^. Naturally derived compounds play a very paramount role in pharmaceutical industry and act as a template for synthetic and semi-synthetic modification. Most of the drugs used in the cancer therapy have been extracted from the natural resources but at the same time separation and purification of these bioactive compounds is the most crucial process^15^. Various extraction techniques such as continuous shaking extraction (CSE), microwave assisted extraction (MAE), Soxhlet extraction (SE), ultrasound assisted extraction (UAE) and steam bath assisted extraction (SBAE) have been used for the isolation of bioactive compound. These enable us in obtaining a better quality and high efficiency of targeted yield from the plant. Hence, one has to optimize the recovery of the metabolites using extraction methods^16,17^.

Response surface methodology (RSM) is a statistical tool usually used in optimization of bioactive compound extraction^17,18^. In RSM, optimized responses, i.e. output variables are controlled by several independent (input) variables and found helpful to cut the number of experimental trials needed to test multiple parameters and their interactions^17,19,20^. Therefore, in present work, RSM with Box-Behnken Design (BBD) was used as a statistical tool for the optimization of cucurbitacin extraction. Diabetes is considered as one of the most severe diseases that lead to a risk of cardiovascular diseases, premature death, kidney failure and depression. It can be effectively managed by using natural α-amylase and α-glucosidase inhibitors (AGIs). Very few synthetic AGIs are available but these have an adverse effect on human beings^21^. This necessitates exploring such type of effective, nontoxic and inexpensive type of AGIs derived from plants.

Plants are rich sources of antioxidants that inhibit or delay the oxidation process by blocking the initiation of oxidizing chain reactions and inhibit formation of hazardous reactive oxygen species (ROS)^22^. Such antioxidant potential may evaluated by several antioxidant assays (DPPH radical scavenging assay, ABTS radical cation scavenging and phosphomolybdate assay). Members of the family *Cucurbitaceae* have proved to be promising antioxidants as reported by earlier workers^2,3,17,22^. In addition, various methods have been employed for the analysis of cucurbitacins using TLC, FTIR, HPLC, LC-MS^17,23^. Hence, the goals of the current study were to optimize extraction of CUI, CUB and CUI + B using HPLC and RSM as a suitable statistical tool. Further, several extraction methods have been evaluated to find out the extraction efficiency for maximizing recovery of cucurbitacins from the fruits. In addition, fruit extracts of *D. palmatus* were studied to investigate *in vitro* antioxidant, anti-diabetic and anticancer potential as well as identification of bioactive metabolites using LC-MS. The present study forms the first report on the optimized extraction of the cucurbitacins, some important bioactivities and other metabolites using LC-MS.

## Results and Discussion

### Optimization of cucurbitacins extraction by BBD

On the basis of preliminary experiments (given in Supplementary Figure S1), actual experimental value of independent variables were fixed as follows: SS ratio (A: 1:20, 1:30, 1:40 g/mL), maceration speed (B: 40, 80, 120 rpm), PS (C: 150, 300, 450 µm), extraction time (D: 30, 60, 90 min) and extraction temperature (E: 40, 50, 60 °C). Interaction between selected factors and optimal conditions were studied using statistical model, i.e. RSM. The design matrix of 46 runs was depicted by employing BBD and their respective responses (CUI, CUB and CUI + B, mg/g DW) are presented in Table A1 (The results are shown in Supplementary Table A1 and Fig. S1).

### Fitting the model

Five independent variables including SS ratio (A), rpm (B), PS (C), time (D) and temperature (E) were investigated and optimized individually using BBD (Table 1). The yield of CUI ranged from 0.0887–2.3450 mg/g DW. Similarly, yield of CUB ranged from 0.0175–1.5840 mg/g DW. The combined yield of both the cucurbitacins CUI + B was noted in between 0.1379–3.8098 mg/g DW. The maximum yield of CUI was observed at 1:30 g/mL SS ratio, 80 rpm, 150 µm PS, 30 min and 50 °C temperature, while CUB and CUI + B showed maximum yield at 1:40 g/mL SS ratio, 60 min extraction time, while rest of the parameters were in line with CUI (Supplementary Table A1). According to multiple regression analysis on the experimental data, the model for the predicted yield of CUI (Y_1_), CUB (Y_2_) and CUI + B (Y_3_) could be expressed by the following quadratic polynomial equations (in the form of coded values):

1 $$\begin{array}{rcl}{Y}_{1}(CUI) & = & 0.837+0.1086{\rm{A}}-0.0772{\rm{B}}-0.4519{\rm{C}}-0.0825{\rm{D}}+0.0961{\rm{E}}\\  &  & -\,0.164{{\rm{A}}}^{2}-0.148{{\rm{B}}}^{2}+0.127{{\rm{C}}}^{2}-0.035{{\rm{D}}}^{2}-0.143{{\rm{E}}}^{2}-0.041{\rm{AB}}\\  &  & -\,0.429{\rm{AC}}-0.030{\rm{AD}}-0.003{\rm{AE}}+0.025{\rm{BC}}-0.052{\rm{BD}}-0.142{\rm{BE}}\\  &  & +\,0.306{\rm{CD}}-0.505{\rm{CE}}-0.105{\rm{DE}}\end{array}$$

2 $$\begin{array}{rcl}{{\rm{Y}}}_{2}({\rm{CUB}}) & = & 0.3058+0.1286{\rm{A}}+0.0193{\rm{B}}-0.3586{\rm{C}}-0.0455{\rm{D}}+0.0580{\rm{E}}\\  &  & +\,0.0386{{\rm{A}}}^{2}+0.0561{{\rm{B}}}^{2}+0.1753{{\rm{C}}}^{2}+0.1714{{\rm{D}}}^{2}+0.0094{{\rm{E}}}^{2}-0.0559{\rm{AB}}\\  &  & -\,0.3989{\rm{AC}}+0.0512{\rm{AD}}-0.0185{\rm{AE}}-0.0389{\rm{BC}}-0.0448{\rm{BD}}+0.1360{\rm{BE}}\\  &  & +\,0.1508{\rm{CD}}-0.2731{\rm{CE}}-0.0859{\rm{DE}}\end{array}$$

3 $$\begin{array}{rcl}{{\rm{Y}}}_{3}({\rm{CUI}}+{\rm{B}}) & = & 1.14+0.2372{\rm{A}}-0.0579{\rm{B}}-0.8105{\rm{C}}-0.1281{\rm{D}}\\  &  & +\,0.1540{\rm{E}}-0.1254{{\rm{A}}}^{2}-0.0920{{\rm{B}}}^{2}+0.3022{{\rm{C}}}^{2}+0.1363{{\rm{D}}}^{2}\\  &  & -\,0.1341{{\rm{E}}}^{2}-0.0973{\rm{AB}}-0.8274{\rm{AC}}+0.0207{\rm{AD}}-0.0217{\rm{AE}}\\  &  & -\,0.0134{\rm{BC}}-0.0973{\rm{BD}}-0.0056{\rm{BE}}+0.4569{\rm{CD}}-0.7785{\rm{CE}}-0.1909{\rm{DE}}\end{array}$$

**Table 1:** Analysis of variance for the fitted quadratic polynomial model for recovery of CUI, CUB, and CUI + B.

Source^a^	CUI					CUB					CUI + B				
	SS	df	MS	F-value	p-value	SS	df	MS	F-value	p-value	SS	df	MS	F-value	p-value
Model	6.92	20	0.3461	2.86	0.0069^**^	4.04	20	0.2021	3.17	0.0036^**^	20.14	20	1.01	3.61	0.0014^**^
A-SS ratio	0.1887	1	0.1887	1.56	0.2231	0.2647	1	0.2647	4.15	0.0524	0.9004	1	0.9004	3.23	0.0844
B-RPM	0.0953	1	0.0953	0.7885	0.3830	0.0060	1	0.0060	0.0935	0.7623	0.0536	1	0.0536	0.1922	0.6649
C-PS	3.27	1	3.27	27.03	< 0.0001^**^	2.06	1	2.06	32.24	< 0.0001**	10.51	1	10.51	37.70	< 0.0001^**^
D-min	0.1090	1	0.1090	0.9018	0.3514	0.0331	1	0.0331	0.5192	0.4779	0.2624	1	0.2624	0.9412	0.3413
E- Temp	0.1476	1	0.1476	1.22	0.2797	0.0538	1	0.0538	0.8425	0.3675	0.3795	1	0.3795	1.36	0.2543
AB	0.0068	1	0.0068	0.0566	0.8138	0.0125	1	0.0125	0.1959	0.6619	0.0379	1	0.0379	0.1358	0.7156
AC	0.7345	1	0.7345	6.08	0.0209*	0.6365	1	0.6365	9.97	0.0041^**^	2.74	1	2.74	9.82	0.0044^**^
AD	0.0037	1	0.0037	0.0307	0.8623	0.0105	1	0.0105	0.1644	0.6886	0.0017	1	0.0017	0.0062	0.9380
AE	0.0000	1	0.0000	0.0003	0.9858	0.0014	1	0.0014	0.0215	0.8845	0.0019	1	0.0019	0.0067	0.9352
BC	0.0026	1	0.0026	0.0215	0.8846	0.0060	1	0.0060	0.0947	0.7608	0.0007	1	0.0007	0.0026	0.9600
BD	0.0110	1	0.0110	0.0912	0.7652	0.0080	1	0.0080	0.1258	0.7258	0.0379	1	0.0379	0.1358	0.7156
BE	0.0802	1	0.0802	0.6631	0.4231	0.0740	1	0.0740	1.16	0.2919	0.0001	1	0.0001	0.0004	0.9834
CD	0.3749	1	0.3749	3.10	0.0905	0.0909	1	0.0909	1.42	0.2438	0.8352	1	0.8352	3.00	0.0958
CE	1.02	1	1.02	8.45	0.0075^**^	0.2984	1	0.2984	4.67	0.0404*	2.42	1	2.42	8.70	0.0068*
DE	0.0441	1	0.0441	0.3649	0.5512	0.0295	1	0.0295	0.4622	0.5028	0.1458	1	0.1458	0.5229	0.4763
A²	0.2348	1	0.2348	1.94	0.1757	0.0130	1	0.0130	0.2040	0.6554	0.1372	1	0.1372	0.4922	0.4894
B²	0.1916	1	0.1916	1.59	0.2196	0.0275	1	0.0275	0.4310	0.5175	0.0739	1	0.0739	0.2652	0.6111
C²	0.1407	1	0.1407	1.16	0.2909	0.2681	1	0.2681	4.20	0.0511	0.7972	1	0.7972	2.86	0.1033
D²	0.0107	1	0.0107	0.0888	0.7682	0.2564	1	0.2564	4.02	0.0560	0.1622	1	0.1622	0.5819	0.4527
E²	0.1797	1	0.1797	1.49	0.2342	0.0008	1	0.0008	0.0121	0.9134	0.1569	1	0.1569	0.5628	0.4601
Residual	3.02	25	0.1209			1.60	25	0.0638			6.97	25	0.2788		
Lack of Fit	2.30	20	0.1149	0.7939	0.6804	1.20	20	0.0599	0.7540	0.7067	5.40	20	0.2701	0.8614	0.6377
Pure Error	0.7238	5	0.1448			0.3973	5	0.0795			1.57	5	0.3136		
Cor Total	9.95	45				5.64	45				27.11	45			

^a^Independent variables, SS- sum of squares; MS – mean square, df – degree of freedom.

*Significant at p-value < 0.05.

**Significant at p-value < 0.01.

**Table 2:** Influence of extraction methods on cucurbitacins (CUI, CUB, and CUI + B) content, antioxidant, and antidiabetic activities.

Source	CUI^a^	CUB^a^	CUI + B^a^	DPPH^b^	ABTS^c^	PMA^d^	α-Amylase^e^	α-Glucosidase^e^
CSE	1.135 ± 0.01^b^	0.584 ± 0.06^b^	1.70 ± 0.28^b^	16.1 ± 0.5^b^	70.1 ± 3.3^ab^	499.1 ± 51.89^bc^	46.55 ± 1.50^b^	48.52 ± 0.39^bc^
MAE	0.760 ± 0.01 ^c^	0.336 ± 0.02^c^	1.08 ± 0.22^c^	16.9 ± 0.7^b^	81.9 ± 0.45^a^	425.4 ± 8.4^c^	68.68 ± 0.66^a^	48.34 ± 0.72^c^
SBAE	1.437 ± 0.03^a^	0.782 ± 0.10^a^	2.17 ± 0.35^a^	25.1 ± 0.1^a^	79.0 ± 12.6^ab^	496.4 ± 10.7^bc^	33.52 ± 1.87^d^	50.60 ± 0.80^b^
UAE	0.723 ± 0.05^d^	0.298 ± 0.01^d^	1.02 ± 0.09^d^	11.3 ± 0.6^c^	14.4 ± 3.2^c^	577.4 ± 9.6 ^a^	44.43 ± 0.11^c^	56.27 ± 0.60^a^

^a^(mg/g DW), ^b^(µg AAE/g), ^c^(µg TE/g), ^d^(µg AAE/g), ^e^% inhibition.

AAE: ascorbic acid equivalents, TE: trolox equivalents.

Values were the means of three replicates ± Standard Error (SE). Mean value with different alphabets in column were showed statistically significant differences (p < 0.05) according to Duncan multiple range test.

The significance of each coefficient was determined by F-test and p-value which are listed in Table 1. Respected variables are known to be more significant when they reflect greater F and smaller p-values^24^. It could be seen that single independent variable, particle size (C) revealed largest effect on the studied responses viz. CUI, CUB and CUI + B (p < 0.01). The results suggested that changing particle size showed remarkable effect on yield of CUI, CUB and CUI + B. The remaining variables like SS ratio (A), rpm (B), time (D) and temperature (E) were found to be statistically insignificant (p > 0.05). Similarly, interaction of parameters was found to be insignificant (p > 0.01) except particle size and temperature (CE) (p < 0.05), and solute solvent ratio and particle size (AC) (p < 0.05) in CUI model. Similarly, particle size and extraction temperature (CE) noted statistically significant in CUB and CUI + B model (p < 0.05). The p-values of all the three models studied were 0.006, 0.003 and 0.001, respectively stated that all the models were significant as well as F values 2.86, 3.17 and 3.61 implied that model was found to be the best fit. Lack of fit was also non-significant (p > 0.05) in all the three models which throw light on significance of model (Table 1). The beneficence of fit of the models was inspected by coefficient of determination (R^2^), which revealed that the explained sample variation was 69.61, 71.70 and 74.29%, respectively. Also, adjusted coefficients of determination (Adj R^2^ = 45.30, 49.06 and 53.73%) were also gratifying to confirm the significance of models.

From the above mentioned Eqs. (1), (2) and (3), it can be summarized that positive coefficient of factor A and E indicated that increase in SS ratio up to threshold level and temperature could bring positive response, whereas a decrease in maceration speed, particle size and extraction time showed positive response on the yield of CUI, as well as CUB. Among the interactions between the variables, responses highly depended on PS, SS ratio and temperature. From the study, it was noted that these three independent variables significantly contributed towards the models.

### Response surface optimization and recovery of cucurbitacins

**Figure 1:**
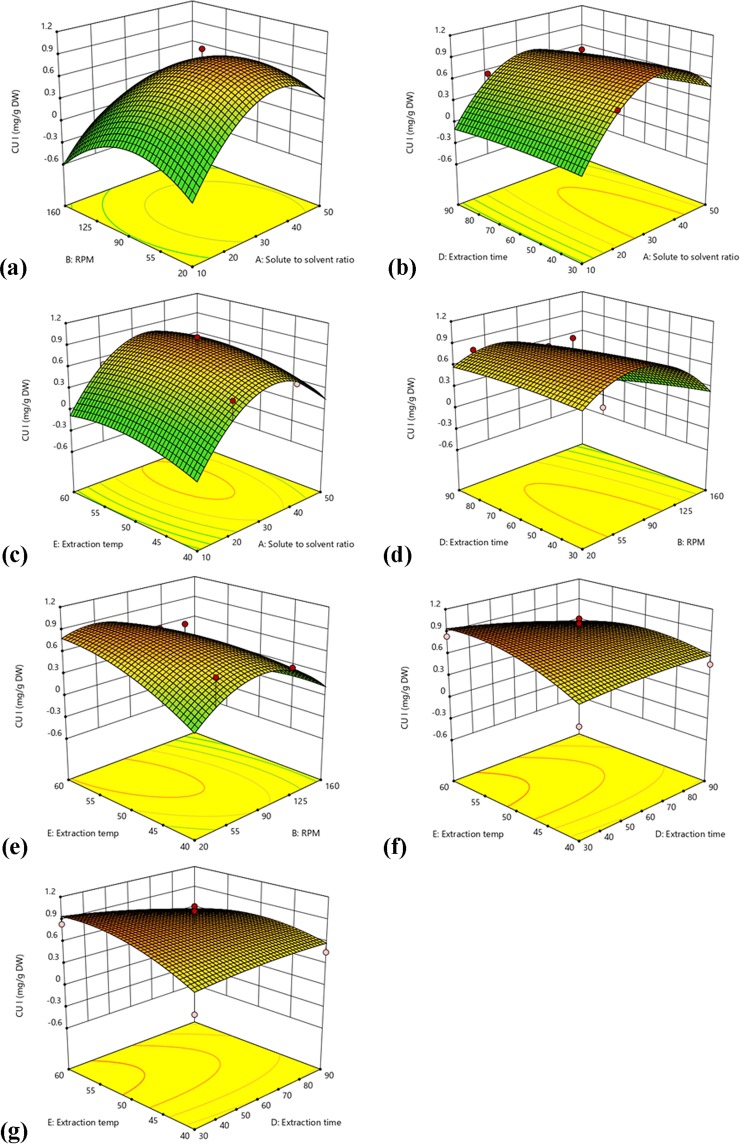
Response surface and counter plots showing the interaction effect of A and B (**a**), A and D (**b**), A and E (**c**), B and D (**d**), B and E (**e**), D and E (**f**), and E and C (**g**) on the extraction yield of CUI.

**Figure 2:**
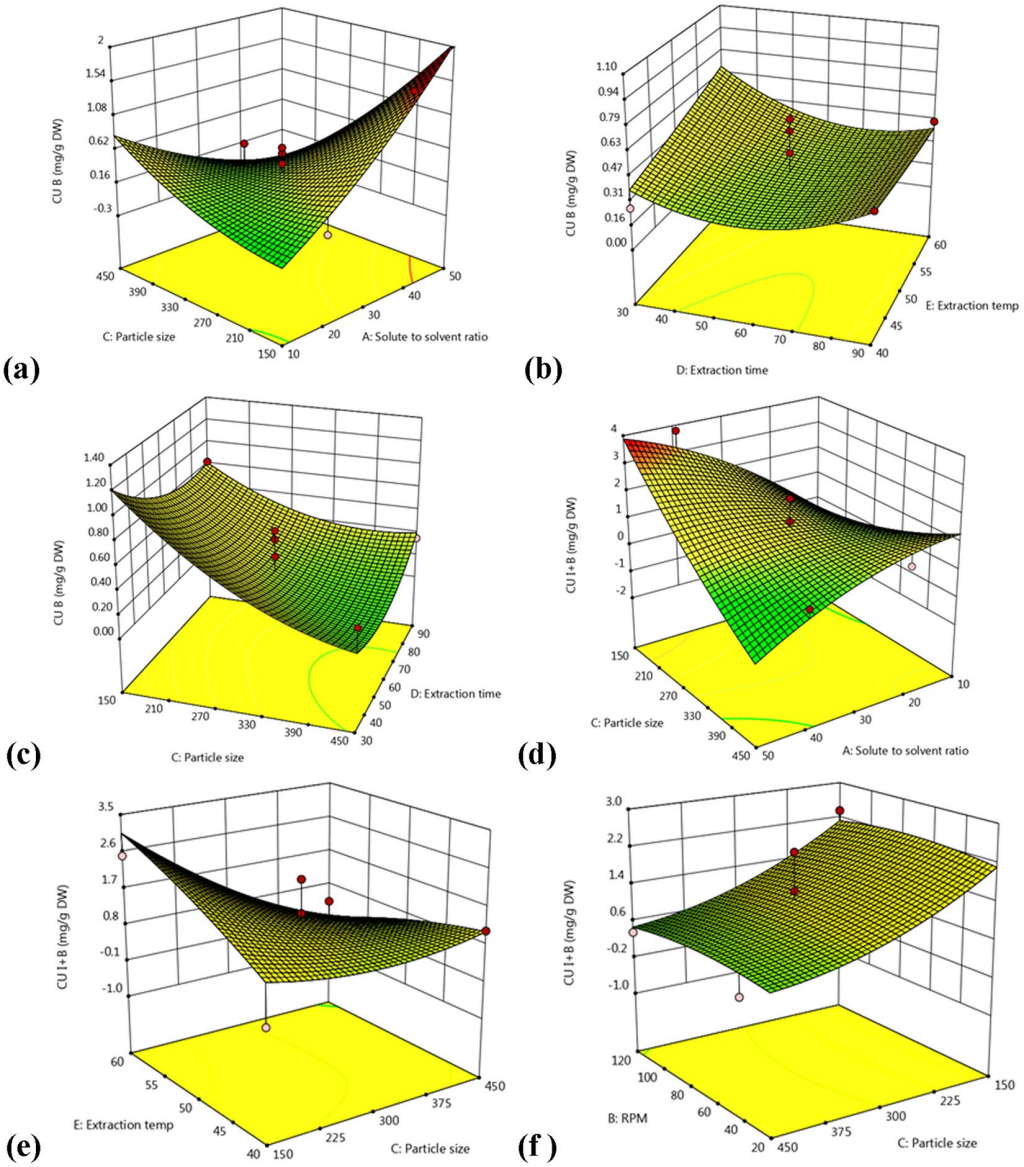
Response surface and counter plots showing interaction effect of A and C (**a**), D and E (**b**), C and D (**c**) on the yield of CUB, and A and C (**d**), C and E (**e**), and B and C (**f**) on the yield of CUI + B.

The relationship between input and output variables was depicted by the representation of three dimensional response surface plots generated by the model (Figs. 1 and 2). Among all the studied input variables (SS ratio, rpm, PS, time and temperature), only two variables within the experimental range were depicted in surface plots and third was kept constant at zero level. The shapes of the contour plots (elliptical or circular) indicated the significance of the interactions between the corresponding variables^25^. The response surface plots for the CUI, CUB and CUI+B were constructed according to their fitted models. Figure 1 represents the plots with one variable kept at a medium level and the other two within the tested ranges. For the determination of optimal level of coded factors, 3D plots were constructed according to Eqs. (1), (2) and (3). Figure 1a showed the effect of SS ratio (A) and rpm (B) on the yield of CUI at fixed PS, time and temperature. An increase in SS ratio from 1:10 to 1:40 g/mL led to an increase in the yield from 0 to 0.5 mg/g DW. Similarly, increase in rpm from 40 to 90 showed positive influence on the yield; however, further increase in SS ratio and rpm showed decline in CUI yield. Moderate volume of solvent was sufficient to complete the extraction of CUI, while very low volume reduces contact of solvent and very high volume dissolves the extractive in solvent resulting in lower yield. This is empirically supported by the significance of SS ratio and rpm observed in the fitted model for CUI. The interaction between extraction time (D) and SS ratio (A) (Fig. 1b) revealed that extraction time in between 30 to 80 min showed slight change in CUI yield (0.6 to 0.8 mg/g DW). However, SS ratio at 1:30 to 1:40 g/mL showed positive influence towards CUI yield. Hence, the extraction time did not play crucial role in CUI extraction and was found insignificant. Figure 1c showed the effect of extraction temperature on CUI yield. At 1:20 g/mL SS ratio, temperature poorly affected on yield, while at 1:30 g/mL SS ratio led to a sharp increase in yield; from 0.4 to 0.8 mg/g DW. The temperature near about 45 to 50 °C and SS ratio 1:30 g/mL significantly influence the yield of CUI. The results agreed with Devendra *et al*.^25^ who reported that temperature and compound yield was directly proportional to each other. In the context of evaluating the effect of rpm on the extraction yield, an increase in rpm from 40 to 80 led to an increase in extraction yield from 0.2 to 0.8 mg/g DW with increasing time. However, further rise in rpm resulted in progressive decline of extraction yield, hence, rpm also found to be a significant factor that positively influenced the yield of CUI (Fig. 1d). Figure 1e depicted the effects of rpm (B) and temperature (E) on the CUI content while all the factors which left behind were invariable. At definite extraction temperature of 45 °C, an increase in rpm from 55 to 110 led to little increase in extraction efficiency (0–0.6 mg/g DW). Similarly, the studied temperature range (55–60 °C) revealed an increase in the extraction yield (0.6–0.8 mg/g DW). The results pointed out that higher temperature (up to 60 °C) was ideal for the extraction of CUI. The interaction between extraction temperature (E) and time (D) (Fig. 1f) showed that at definite time (45–85 min), there was a steady increase in CUI content (0.6 mg/g DW), which pointed out that time has no significant role. At the same time, with increase in temperature (from 40 to 60 °C), there was sharp increase in the CUI (0–0.9 mg/g DW). It indicated that temperature significantly influences the yield of CUI (Fig. 1f).

The influence of particle size (C) on the extraction yield (Fig. 1g) revealed that an increase in particle size from 150 to 450 µm led to sharp decrease in the yield from 1.5–0.6 mg/g DW. Therefore, particle size and yield of CUI were found inversely proportional to each other. Decrease in particle size resulted into greater surface area of particles that came in contact with solvent which subsequently enhance the leaching of CUI in the medium. In additon, decreasing in particle size and gradual increase in temperature also positively affected the extraction rate. The maximum yield was found at 150 µm PS at 60 °C. Therefore, particle size was noted to be the most significant variable for maximum CUI extraction.

Similarly, surface plots were constructed for determining effect of extraction parameters on extraction of CUB and CUI+B. The Fig. 2a showed effect of particle size (C) and SS ratio (A) on a yield of CUB. Increase in SS ratio from 1:10 to 1:50 g/mL resulted in the increase of yield of CUB from 0.017 to 2 mg/g DW. Therefore, combination of these two factors was highly significant for the extraction of CUB. The interaction between time (D) and temperature (E) revealed that temperature positively affects the yield of CUB (0.3 to 0.8 mg/g DW) at 30 min extraction time (Fig. 2b). In addition, minimal extraction time from 30 to 40 min showed significant effect, while further increase in extraction time up to 60 min decreased CUB content. Figure 2c represents mutual interaction between particle size (C) and extraction time (D). The smaller particle size and shorter extraction period positively influence the yield of CUB (up to 1.20 mg/g DW) but further increase in time as well as particle size led to loss of yield up to 0.8–0.2 mg/g DW. Therefore, a combination of short extraction time and the particle size (150 µm) revealed the highest CUB content.

The surface plots of the CUI + B content represented an interactive effect between SS ratio (A) and PS (C) (Fig. 2d). Similar to other interactions, lower particle size exhibited positive effect on extraction of both the cucurbitacins. From the Fig. 2e, it was noticed that interactive effect between PS (C) and temperature (E) demonstrated the positive influence of smaller particle size and higher temperature on yield of cucurbitacins (1.7 to 2.7 mg/g DW). The interactions of maceration speed and particle size are represented in Fig. 2f. We pointed out that the lower particle size was statistically significant for the optimized extraction, but maceration speed did not show considerable effect on the yield of both the cucurbitacins under investigation (Fig. 2).

### Validation of the predictive model

Optimal levels obtained from the fitted model for independent variables were 1:40 g/mL, 40 rpm, 150 µm PS, 30 min and 60 °C for CUI, whereas only factor maceration speed changes for CUB and CUI + B which was 120 rpm for CUB and 80 rpm for CUI + B and rest of remaining all factors were similar like CUI. The predicted yield obtained by above condition of CUI, CUB and CUI + B was 2.352, 2.407 and 4.759 mg/g DW, respectively. Similarly, three experiments were carried out under suggested optimal conditions and results showed that extraction yield of CUI, CUB and CUI + B were 2.299, 2.389 and 4.63 mg/g DW, respectively. By comparing experimental and predicted values given by Design-Expert software, it was noticed that predicted values were close to actual value, therefore, the proposed optimized parameters in the present work were found highly reliable for the recovery of cucurbitacins. On the basis of our findings, maceration speed was the most crucial variable for the yield of different cucurbitacins. The recovery of cucurbitacins was dependent on varied levels of maceration speed wherein ≤40, 80 and 120 rpm showed higher content of CUI, CUI + B and CUB, respectively.

### Comparison of extraction methods

**Figure 3:**
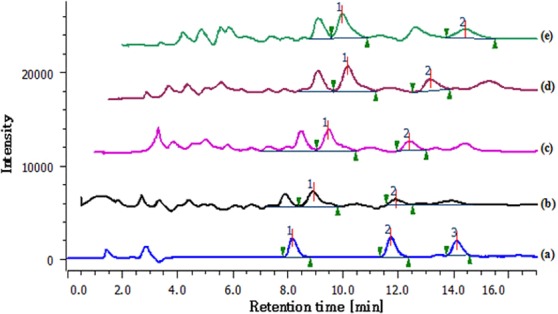
HPLC chromatogram of standards of CUI (**1**), CUB (**2**), and CUE (**3**) (**a**), as well as fruit extract of *D. palmatus* obtained by UAE (**b**), SBAE (**c**), MAE (**d**) and CSE (**e**).

The extraction efficiency of various extraction methods viz. CSE, UAE, MAE and SBAE for maximizing the recovery of cucurbitacins was evaluated and results are presented in Table 2 and Fig. 3. Conventional methods like CSE and SBAE have been traditionally used for extraction of terpenes, phenolics and other antioxidant compounds^16,17^.

Analysis of extracts obtained from different extraction methods was done by comparing peak area of standards CUI and CUB, with the chromatogram of obtained extracts. The comparative account of cucurbitacin content from the samples is given in Fig. 3 and Table 2. The highest CUI, CUB and CUI + B (1.437 ± 0.03, 0.782 ± 0.10 and 2.17 ± 0.35 mg/g DW, respectively) were studied from the extract of SBAE (Table 2), whereas lowest CUI, CUB and CUI + B (0.7228 ± 0.005, 0.298 ± 0.001 and 1.02 ± 0.09 mg/g DW, respectively) contents were found in UAE. Similarly, other extraction methods also showed dependable cucurbitacin content and showed the content was higher in SBAE > CSE > MAE > UAE. Among all the extraction methods employed, SBAE at 50 °C indicated that temperature played a crucial role in highest recovery of the cucurbitacins. The extraction depends on penetration and interaction of solvent with the plant materials, solubility, and diffusion of the compounds in the medium and harvesting of the targeted solute. Different extraction methods exhibited varied degree of solubility and diffusivity that accelerated the yield of the cucurbitacins or hinder the content by forming undesirable impurities and decomposition of the thermostable compounds^17,26^.

## Antioxidant Activities

Antioxidants are the molecules which inhibits or delay the oxidative damage caused by reactive oxygen species (ROS)^2,3^. Generally, antioxidant potential can be evaluated by DPPH, ABTS, FRAP and phosphomolybdate assay. In the present investigation, extracts obtained by various extraction methods were subjected to evaluation of their antioxidant potential and the results are demonstrated in Table 2. SBAE showed the highest DPPH activity (25.1 ± 0.1 µg AAE/g DW) while least in UAE (11.3 ± 0.6 µg AAE/g DW). However, extracts evaluated from CSE and MAE did not show significant difference in DPPH radical scavenging activity. The maximum ABTS radical scavenging activity was noted from MAE (81.9 ± 0.45 µg TE/g DW) and minimum in the UAE (14.4 ± 3.2 µg TE/g DW). All the extracts showed promising phosphomolybdate activity wherein the highest was found in UAE (577.4 ± 9.6 µg AAE/g DW). Variable response for the antioxidant activities may on account of presnece of other phytochemicals present in it. Several researchers reported antioxidant potential of *D. palmatus* and other cucurbits^3,22,27^.

## Anti-Diabetic Activity

### α-amylase inhibition activity

Diabetes is one of the main systematic illnesses throughout the world. Alpha-amylase inhibitors (AAI) are playing a significant role in the treatment of diabetes^28^. The α-amylase enzyme is responsible for breakdown of oligo and/or disaccharide into monosaccharides. Inhibitor of these enzymes delays carbohydrate digestion, causing a marked decrease in glucose absorption and this decreased level is responsible for decreasing the post-prandial hyperglycemia^29^. The extract prepared from MAE exhibited the highest inhibition of α-amylase (68.68 ± 0.66%), while least was recorded in SBAE (33.52 ± 1.87%) (Table 2). The moderate inhibition was recorded from CSE and UAE extracts (46.55 ± 1.50, 44.43 ± 0.11%, respectively). Similar results have been reported from *Citrullus colocynthis*^30^. In contrast, aqueous extract of *Kedrostis africana* tuber represented 31.64 ± 1.11% α-amylase inhibitory activity at 200 mg/mL extract concentration^31^.

### α-glucosidase inhibitory activity

The deficiency of insulin secretion causes type 2 diabeties which results in hyperglycemia, physical and mental burden and subsequnt increase in the rate of mortality and disability^28^. α-Glucosidase enzyme is responsible for release of glucose by hydrolyzing linear and branched iso-maltose oligosaccharides which cause postprandial hyperglycemia. Therefore, identifying and characterizing inhibitor of α-glucosidase is very important^32^. In present investigation, the extract prepared by UAE exhibited the highest response (56.27 ± 0.60%), and the lowest by MAE (48.34 ± 0.72%). Similarly, SBAE and CSE demonstrated reliable inhibitory activity (50.60 ± 0.80, 48.52 ± 0.39%, respectively) (Table 2). Our results are in good agreement with Unuofin *et al*.^31^ who reported that ethanol extract of *Kedrostis africana* tubers revealed 54.30 ± 0.79% α-glucosidase inhibitory activity.

## Anticancer Activity

Anticancer activity of *D. palmatus* fruit extract was evaluated by SRB (sulphorhodamine B) assay. SRB assay is sensitive and high-throughput method for evaluating cytotoxic activity against cancer cell lines. The assay is independent from cell metabolic activity which is not obstructed by test compounds and very simple to perform^31,33^. The GI_50_, TGI (cytostatic activity) and LC_50_ (cytotoxic activity) values were calculated for each extract against two cancer cell lines, viz. breast and colon cancer cell lines MCF-7 and HT-29. Details of complete cell line activity are represented in Supplementary Table A2 and Fig. S2. In this experiment, fruit extract showed significant inhibitory activity in cancer cell growth. The growth inhibition percentage of both cancer cell lines was found to be increasing with increasing concentration of the extracts. The highest anticancer activity towards MCF-7 cell line was shown by fruit extract of *D. palmatus* (GI_50_ < 10, TGI 5.14, LC_50_ 44.27 µg/mL). Standard adriamycin revealed anticancer activity (GI_50_ < 10, TGI-104.27, LC_50_ 385.17 µg/mL) which was comparable with the tested fruit extracts. Similarly, fruit extract also exhibited efficient anticancer activity against HT-29 cell line (GI_50_ < 10, TGI 46.88, LC_50_ 68.31 µg/mL). However, adriamycin showed high anticancer activity (GI_50_ < 10, TGI 0.880, LC_50_ 63.20 µg/mL) against HT-29 cell line. Abdel-Salam *et al*.^34^ reported that leaves of *Luffa cylindrica* possess anticancer activity against cancer stem cells CD44+/24− in breast cancer patients (85.18 ± 2.21%). Efficacy of CUB and CUI to inhibit breast and colon cancer has been also reported earlier^35,36^. Cai *et al*.^37^ reviewed the synergistic effect of the CUB and several other drugs (gemcitabine, docetaxel, methotrexate and cisplatin) against cancer cell lines. In the present study, fruit extract showed CUI, CUB, and other potent metabolites that could be responsible for the promising anticancer activities. These results highlighted that the fruits of *D. palmatus* can be a novel source of anticancer drug.

### Identification of major metabolites by LC-MS

The LC-MS analysis of *D. palmatus* fruits allowed identification of total eleven major compounds from different categories. Entire list of these compounds can be found in Supplementary Table A3. Our study revealed the presence of these detected compounds including CUI and CUB. The mass spectra of CUI and CUB showed the characteristic protonated ion at m/z 497.2855 and 540.696 ([M + H]^+^), respectively, which appeared at retention time 15.90 and 15.99 min. Beta-hederin from saponin category showed highest mass about 752.491 at m/z 734.45 ([M + H]^+^). Delmas *et al*.^38^ reported anti-leishmanial activity from the saponin. Two steroids, (digoxigenin monodigitoxoside and minabeolide-8) have been reported from the *Digitalis purpurea* and *Minabea* species, respectively, having cytotoxic and anti-inflammatory activities^39,40^. A single alkaloid deserpidine (molecular mass 578.26 g/mol) is an ester alkaloid drug isolated from *Rauvolfia canescens* exhibited antihypertensive properties^41^. Luteolin 7-(2′′-p-coumaroylglucoside) (594.134 g/mol), a flavonoid known to be anticarcinogenic have been studied from *Ophiorrhiza mungos*^42^. Two cardiac glucosides, viz. digitalose and bufadienolide detected in the present study have been reported earlier as a cardioprotective^43^. Similarly, bufadienolides exhibited wide range of biological properties includes cytotoxic, antitumor and cardiotonic activities^44^. Caffic acid (molecular mass 342.095 g/mol) was also noted which is helpful for avoiding oxidative deterioration of food and maintain food colors, favors and nutritional values, as well as other activities like antioxidant, anti-mutagenic, anti-tumor, and anti-obesity were reported^45^. The compounds with the lowest molecular mass (125.05 g/mol) i.e. isovaleric acid reported in the current study, belong to fatty acid category. This compound is known for unpleasant odor, produced by skin bacteria, and it is found in valerian plant^46^. In valerian extracts, this acid shows anticonvulsant activity^47^.

## Conclusion

In the present study, ideal model for maximum extraction of cucurbitacin (I, B and I + B) from *D. palmatus* fruits was successfully studied using response surface methodology (RSM) with Box-Behnken design (BBD). The study recommended *D. palmatus* fruit extraction with chloroform at 1:30 g/mL SS ratio, 80 rpm, 150 µm particle size, 30 min time and 50 °C temperature, found to be the best for maximizing the recovery of CUI (2.3450 ± 0.1686 mg/g DW). Significantly higher CUB (1.5840 ± 0.15 mg/g DW) and CUI + B (3.8021 ± 0.31 mg/g DW) were recorded using 1:40 g/mL SS ratio and 60 min extraction time, while keeping remaining parameters as mentioned in CUI. Several extraction methods such as CSE, SBAE, MAE and UAE were studied wherein the highest cucurbitacins content was reported from SBAE. Antioxidant potential of each extract was dependent on extraction method wherein the highest DPPH, ABTS and phosphomolybdate activities were recorded from SBAE, MAE and UAE, respectively. The recorded variation could be due to the diverse antioxidant profile of each extract along with cucurbitacins obtained from various extraction methods. MAE and UAE were efficient to extract the inhibitors of enzymes α-amylase and α-glucosidase that can be used to manage diabetes. The extract tested against cancer cell lines revealed that *D. palmatus* fruit exhibited promising anticancer activities that will assist us to pave a path for the development of a novel anticancer drug.

## Materials and Methods

### Chemicals and reagents

Analytical and HPLC grade solvents (chloroform, methanol, ethanol, hexane, acetone, ethyl acetate, acetonitrile and water) were purchased from Sisco research laboratory Pvt. Ltd., India. Standard cucurbitacin I (CUI) and cucurbitacin B (CUB) were purchased from Sigma Aldrich (St. Louis, Mo, USA).

### Collection of plant material and preparation of extract

The fruits of *D. palmatus* L. were collected from Panhala, Maharashtra, India in August 2017. The location lies between N16°48′540′′ latitude and E74°07′757′′ longitude. The fruits were oven dried for 72 h at 60 ^°^C, ground into fine powder (300 µm) and extracts were obtained by continuous shaking of fruit powder (1 g) with respective solvents. Extracts were then filtered with Whatman No. 1 filter paper, concentrated and dissolved in 1 mL methanol, filtered through 0.2 µm nylon filter (HiMedia, India) and used for HPLC analysis.

### Optimization of cucurbitacins extraction using RSM

After a preliminary experiment (Supplementary data), extraction of cucurbitacins was further optimized using RSM. RSM with five factors and three levels in Box-Behnken design (BBD) was used to optimize variables for extraction of cucurbitacins. On the basis of outcome, the independent variables were SS ratio (A: 1:20–1:50 g/mL), maceration speed (B: 80–200 rpm), particle size (C: 150–600 µm), time (D: 30–120 min) and temperature (E: 30–60 ^°^C), and the dependent (response) variables were yield of CUI, CUB and CUI + B. All the variables were modified as –1, 0 and 1 for the evaluation of factors. The BBD was generated by Design-Expert software trial ver. 11. According to this design, total 46 experiments were performed (Supplementary Table A1). A second order polynomial equation, which relates the measured response to independent variables, was used:

$$Y={{\beta }}_{0}\,+\,\mathop{\sum }\limits_{{i}=0}^{{k}}{{\beta }}_{{i}}{{X}}_{{i}}+\mathop{\sum }\limits_{{i}=0}^{{k}}{{\beta }}_{{ii}}{{X}}_{{i}}^{2}+\mathop{\sum }\limits_{{i}=0}^{{k}}\mathop{\sum }\limits_{{j}=0}^{{k}}{{\beta }}_{{ij}}{{X}}_{{i}}{{X}}_{{j}}$$

where Y is the yield, β_0_ is the intercept term, β_i_ is the linear coefficient, β_ii_ is the quadratic coefficients, β_ij_ is the interaction coefficients, and X_i_ and X_j_ are uncoded independent variables.

The contour and surface plots were generated by keeping other response variables at its optimal level and plotting other two independent factors with the responses. After treatment, the extracts were prepared as described earlier and used for HPLC analysis.

## Extraction of Cucurbitacins By Various Extraction Methods

Different extraction methods such as continuous shaking extraction (CSE), microwave assisted extraction (MAE), ultrasound assisted extraction (UAE) and steam bath assisted extraction (SBAE) were employed to study variable response of the cucurbitacins extraction according to method reported earlier^17^. For the extraction of cucurbitacins using all the extraction techniques, 1 g of the fruit powder and chloroform as a solvent were used.

### Continuous shaking extraction (CSE)

CSE was carried out using a shaker incubator (Allied Scientific Products, India) at controlled conditions (120 ± 2 rpm; 30 ± 1 °C) for the periods of 15, 30, 60 and 90 min.

### Microwave assisted extraction (MAE)

MAE was carried out in microwave oven (Haier, India). Fruit powder was taken in 250 mL conical flask containing 30 mL of solvent and extraction was studied at varied time intervals (30, 60, 120 and 180 s).

### Ultrasound-assisted extraction (UAE)

UAE was performed using an ultrasonic bath (Rivotek, India). Samples were exposed for 15, 30, 45 and 60 min at room temperature keeping other parameters constant as mentioned earlier.

### Steam bath assisted extraction (SBAE)

SBAE was carried on a stirred thermal water bath (Equitron, India) at a varying temperature (30, 40, 50 and 60 °C) and fixed time of 60 min.

All the extracts were evaporated, dissolved in 1 mL methanol, filtered using 0.22 µm membrane filter and used for further analysis.

### HPLC conditions

The HPLC apparatus was equipped with quaternary pump, autosampler, and UV detector (Jasco, Japan). The separation of cucurbitacins was achieved on Hiber C18 column (250x4.6 mm, 5 μm) and using published protocol with minor modifications^48^. The mobile phase with solvents acetonitrile, water and methanol (32:35:33, v/v/v) was used. The flow rate was 1.0 mL/min with 20 μL injection volume. Peaks were monitored at 230 nm up to 40 min and content of cucurbitacins were expressed in miligram per gram of dry weight (mg/g DW).

## Antioxidant Activities

### DPPH radical scavenging assay

Extracts were analyzed to evaluate DPPH (1,1-diphenyl-2-picrylhydrazyl) radical scavenging activity as per our earlier protocols^2,3,17,22,28^. The working solution DPPH reagent (1 mL) was mixed with 500 μL of plant extract (1 mg/mL). The mixed reaction mixtures were incubated in dark for 30 min, and absorbance was read at 517 nm.

### ABTS radical scavenging assay

ABTS [2,2′-azino-bis (3-ethylbenzothiazoline-6-sulphonic acid)] radical cation decolorization assay was used with slight modifications^2,3,17,22,28^. ABTS solution (1 mL) was treated with 100 µL of plant extract and incubated at 30 °C. Trolox was used as a standard. After 30 min, absorbance was read at 730 nm using ethanol as blank.

### Phosphomolybdate assay (PMA)

PMA assay was performed as per earlier protocols^3,17,22,28^, in which fruit extract (300 μL) was treated with 1 mL phosphomolybdate reagent and incubated at 95 °C for 90 min, cooled and absorbance was recorded at 765 nm. Ascorbic acid was used to obtain a calibration curve.

## Anti-Diabetic Activity

### α-amylase inhibition activity

The α-amylase inhibition was studied using standard protocols with the minor changes^17,28^. Briefly, a plant extract was mixed with α-amylase solution (70 µL from 18 U/mL) and diluted to 1 mL using 0.02 M sodium phosphate buffer (pH 6.9). The reaction mixture was incubated at 25 °C for 10 min, 500 µL of 1% starch solution was added to it and kept at 25 °C for 30 min. Post incubation, reaction was stopped by addition 0.5 mL dinitrosalicylic acid reagent. Reaction mixtures were incubated at 100 °C for 5 min, cooled, diluted with double distilled water and absorbance was taken at 540 nm. The control was also produced as mentioned earlier wherein instead of extract sodium phosphate buffer was added. Acarbose was used as a positive control and the activity was calculated on a percent basis.

### α-glucosidase inhibition activity

α-Glucosidase inhibition was carried as per the published protocols^17,28^. The fruit extract was treated with 100 µL α-glucosidase solution (U/mL) diluted to 500 µL by adding 0.1 M phosphate buffer (pH 6.9). After 5 min incubation at 25 °C, 100 µL p-nitrophenyl-α-D-glucopyranoside (5 mM) solution was added to the reaction mixture and incubated at 25 °C for 10 min. Further, 0.1 M Na_2_CO_3_ (1 mL) was added and absorbance was measured at 405 nm. The control was prepared without any extract and volume adjusted by adding buffer solution. Acarbose was used as a positive control and activity was expressed on a percent basis.

## Anticancer Activity

In the present study, breast (MCF-7) and colon (HT-29) cancer cell lines (procured from National Cancer Institute, USA) were used to assess the anticancer potential^49,50^. The cells grown on RPMI 1640 medium were inoculated into 96 well microtiter plates (100 µL/well). The plates were kept at controlled conditions (24 h, 37 °C, 5% CO_2_, 95% air and 100% relative humidity). Samples dissolved in DMSO (mg/mL) were serially diluted with the medium (10–80 μg/mL). Further, all the plates were kept for 48 h. Then the assay was terminated by cold trichloroacetic acid (TCA). All the cells were fixed *in situ* by adding with 50 µL of TCA. The supernatant was discarded and the plates were washed with distilled water and air dried. SRB solution (50 µL) was added to each well and maintained at room temperature. The surplus dye was washed with 1% acetic acid and all the plates were air dried. Excess stain was removed by 10 mM trizma base and the absorbance was read at a wavelength of 540 nm and 690 nm. Percent growth was calculated and results were expressed as % control growth, TGI and LC_50_.

### Validation of CUI, CUB and detection of other metabolites by LC-MS method

HPLC System (Agilent, USA) equipped with an autosampler, binary pump, thermostated column compartment and 6550 iFunnel Q-TOF with jetstream electrospray ionization source in positive mode was used as per our earlier report^22^. Zorbax eclipse C18 column (2.1 × 150 mm, 5 µm) at 25 °C was used for the separation. The mobile phase consisting water (A) and acetonitrile (B) modified with 0.1% (v/v) formic acid was used at 0.2 mL/min flow rate. An analysis was done by gradient elution (2 min 5% B, 95% up to 25 min, 26–30 min 5% B). A sample extract (8 µL) was used for the analysis and mass spectrometer was operated from m/z 50–1000. The N_2_ was used as nebulizer, drying and collision gas. The nebulizer gas was used at 45 psig; fragmentor voltage 175 V and capillary voltage was set to 3.5 kV. All the data was analyzed using the mass hunter qualitative analysis software package (Agilent Technologies).

### Statistical analysis

All experiments were performed in triplicate and values were represented as mean ± standard error. Data from extraction methods and their bioactivities were subjected to analysis of variance (ANOVA) using SPSS software.

## Supplementary information

Supplementary Information.

## References

[1] Renner SS, Pandey AK (2013). The Cucurbitaceae of India: accepted names, synonyms, geographic distribution and information on images and DNA sequences. PhytoKeys..

[2] Attar UA, Ghane SG (2017). Proximate composition, antioxidant activities and phenolic composition of *Cucumis sativus* forma *hardwickii* (Royle) W. J. de Wilde & Duyfjes. Int. J. Phytomed..

[3] Attar UA, Ghane SG (2017). Phytochemicals, antioxidant activity and phenolic profiling of *Diplocyclos palmatus* (L.) C. Jeffery. Int. J. Pharm. Pharm. Sci..

[4] Chauhan NS, Dixit VK (2010). Effects of *Bryonia laciniosa* seeds on sexual behavior of male rats. Int. J. Impot. Res..

[5] Chen X (2012). Biological activities and potential molecular targets of cucurbitacins: a focus on cancer. Anti-Cancer Drug..

[6] Zhou Y (2016). Convergence and divergence of bitterness biosynthesis and regulation in Cucurbitaceae. Nat. Plants..

[7] Blaskovich MA (2003). Discovery of JSI-124 (cucurbitacin I), a selective Janus kinase/signal transducer and activator of transcription 3 signaling pathway inhibitor with potent antitumor activity against human and murine cancer cells in mice. Cancer Res..

[8] Wakimoto N (2008). Cucurbitacin B has a potent antiproliferative effect on breast cancer cells *in vitro* and *in vivo*. Cancer Sci..

[9] Chan KT (2010). Cucurbitacin B induces apoptosis and S phase cell cycle arrest in BEL-7402 human hepatocellular carcinoma cells and is effective via oral administration. Cancer Lett..

[10] Safarzadeh E, Shotorbani SS, Baradaran B (2014). Herbal medicine as inducers of apoptosis in cancer treatment. Adv. Pharm. Bull..

[11] Kamatou GPP (2008). Antimalarial and anticancer activities of selected South African *Salvia* species and isolated compounds from. S. radula. S. Afr. J. Bot..

[12] Loman BR, Jordan KR, Haynes B, Bailey MT, Pyter LM (2019). Chemotherapy-induced neuroinflammation is associated with disrupted colonic and bacterial homeostasis in female mice. Sci. Rep..

[13] Graça VC (2019). Isolation of secondary metabolites from *Geranium molle* L. with anticancer potential. Ind. Crops. Prod..

[14] Sun J (2005). Cucurbitacin Q: a selective STAT3 activation inhibitor with potent antitumor activity. Oncogene..

[15] Silva IT (2016). Cytotoxic effects of natural and semisynthetic cucurbitacins on lung cancer cell line A549. Invest. New Drugs..

[16] Upadhya V, Pai SR, Hegde HV (2015). Effect of method and time of extraction on total phenolic content in comparison with antioxidant activities in different parts of *Achyranthes aspera*. J. King Saud. Uni. Sci..

[17] Attar UA, Ghane SG (2018). Optimized extraction of anti-cancer compound - cucurbitacin I and LC–MS identification of major metabolites from wild Bottle gourd (*Lagenaria siceraria* (Molina) Standl.). S. Afr. J. Bot..

[18] Dhanani T, Shah S, Gajbhiye NA, Kumar S (2017). Effect of extraction methods on yield, phytochemical constituents and antioxidant activity of *Withania somnifera*. Arabian J. Chem..

[19] Liyana-Pathirana C, Shahidi F (2005). Optimization of extraction of phenolic compounds from wheat using response surface methodology. Food Chem..

[20] Arun VV (2017). Multi-response optimization of *Artemia* hatching process using split-split-plot design based response surface methodology. Sci. Rep..

[21] Chang SK, Alasalvar C, Shahidi F (2016). Review of dried fruits: phytochemicals, antioxidant efficacies, and health benefits. J. Funct. Food..

[22] Patel SB, Attar UA, Ghane SG (2018). Antioxidant potential of wild *Lagenaria siceraria* (Molina) Standl. Thai J. Pharm. Sci..

[23] Cai C (2019). Extraction and antioxidant activity of total triterpenoids in the mycelium of a medicinal fungus, *Sanghuangporus sanghuang*. Sci. Rep..

[24] Atkinson, A. C. & Donev, A. N. Optimum experimental designs. Oxford Statistical Science Series. 8. Vol. 8. Clarendon Press, Oxford. (1992).

[25] Jin L (2012). Characterization and antioxidant activity of a polysaccharide extracted from *Sarcandra glabra*. Carbohyd. Polym..

[26] Zhang QW, Lin LG, Ye WC (2018). Techiniques for extraction and isolation of natural products: a comprehensive review. Chin. Med..

[27] Misra A (2017). Simultaneous-HPLC quantification of phenolic acids in traditionally used ayurvedic herb *Diplocyclos palmatus* (L.) Jeffry. Pharmacogn. J..

[28] Ghane SG, Attar UA, Yadav PB, Lekhak MM (2018). Antioxidant, anti-diabetic, acetylcholinesterase inhibitory potential and estimation of alkaloids (lycorine and galanthamine) from *Crinum* species: An important source of anticancer and anti-Alzheimer drug. Ind. Crops. Prod..

[29] Safamansouri H (2014). α-Amylase inhibitory activity of some traditionally used medicinal species of Labiatae. J. Diabetes. Metabol. Disord..

[30] Ramzi S, Sahragard A (2013). A lectin extracted from *Citrullus colocynthis* L. (Cucurbitaceae) inhibits digestive α-amylase of *Ectomyelois ceratoniae* Zeller (Lepidoptera: Pyralidae). J. Entomolo. Acarol. Res..

[31] Unuofin JO, Otunola GA, Afolayan AJ (2018). *In vitro* α-amylase, α -glucosidase, lipase inhibitory and cytotoxic activities of tuber extracts of *Kedrostis africana* (L.) Cogn. Heliyon..

[32] Zhang L (2011). Grape skin extract inhibits mammalian intestinal a-glucosidase activity and suppresses postprandial glycemic response in streptozocin-treated mice. Food Chem..

[33] Houghton P (2007). The sulphorhodamine (SRB) assay and other approaches to testing plant extracts and derived compounds for activities related to reputed anticancer activity. Methods..

[34] Abdel-Salam IM, Abou-Bakr AA, Ashour M (2019). Cytotoxic effect of aqueous ethanolic extract of *Luffa cylindrica* leaves on cancer stem cells CD44+/24- in breast cancer patients with various molecular sub-types using tissue samples *in vitro*. J. Ethnopharmacol..

[35] Kim HJ, Park JHY, Kim JK (2014). Cucurbitacin-I, a natural cell-permeable triterpenoid isolated from Cucurbitaceae, exerts potent anticancer effect in colon cancer. Chem. Biol. Interact..

[36] Gupta P, Shrivastava SK (2014). Inhibition of Integrin-HER2 signaling by Cucurbitacin B leads to *in vitro* and *in vivo* breast tumor growth suppression. Oncotarget..

[37] Cai Y (2015). Cucurbitacins: A Systematic Review of the Phytochemistry and Anticancer Activity. Am. J. Chin. Med..

[38] Delmas F (2000). Antileishmanial activity of three saponins isolated from Ivy, α-Hederin, β-Hederin and Hederacolchiside A_1_, as compared to their action on mammalian cells cultured *in vitro*. Planta Med..

[39] Yeh JY, Huang WJ, Kan SF, Wang PS (2001). Inhibitory effects of digitalis on the proliferation of androgen dependent and independent prostate cancer cells. J. Urol..

[40] Chao CH (2011). Paraminabeolides AF, cytotoxic and anti-inflammatory marine withanolides from the soft coral *Paraminabea acronocephala*. J. Nat. Prod..

[41] Lakshmi SRA, Madawela G (2001). Alkaloids from. Rauvolfia canescens. Pharm. Biol..

[42] Baskar AA, Ignacimuthu S, Michael GP, Al Numair KS (2011). Cancer chemopreventive potential of luteolin-7-O-glucoside isolated from *Ophiorrhiza mungos* Linn. Nutr. Cancer..

[43] Fujii Y, Ikeda Y, Yamazaki M (2006). Quantitative determination of digitalis glycosides in *Digitalis purpurea* leaves by reversed-phase thin-layer chromatography. J. Liq. Chromatogr..

[44] Gao H, Popescu R, Kopp B, Wang Z (2011). Bufadienolides and their antitumor activity. Nat. Prod. Rep..

[45] Yamada J, Tomita Y (1996). Antimutagenic activity of caffeic and related compounds. Biosci. Biotechnol. Biochem..

[46] Ara K (2006). Foot odor due to microbial metabolism and its control. Can. J. Microbiol..

[47] Patocka J, Jakl J (2010). Biomedically relevant chemical constituents of *Valeriana officinalis*. J. Appl. Biomed..

[48] Fiori GML (2017). Development and validation of a quantification method for cucurbitacins E and I in rat plasma: Application to population pharmacokinetic studies. J. Pharma. Biomed. Anal..

[49] Vichai V, Kirtikara K (2006). Sulforhodamine B colorimetric assay for cytotoxicity screening. Nat. Protoc..

[50] Skehn P (1990). New colorimetric cytotoxicity assay for anticancer drug screening. J. Natl. Cancer Inst..

