# Supplementary material for: Efficient extraction of cucurbitacins from Diplocyclos palmatus (L.) C. Jeffrey: Optimization using response surface methodology, extraction methods and study of some important bioactivities
Source: Sci Rep. 2020 Feb 7;10:2109. doi: 10.1038/s41598-020-58924-5 (PMC7005863; doi:10.1038/s41598-020-58924-5)
Supplement: Supplementary file 1 — Supplementary Information. [file 41598_2020_58924_MOESM1_ESM.pdf]

Supplementary information

Efficient extraction of cucurbitacins from *Diplocyclos palmatus* (L.) C. Jeffrey: Optimization using response surface methodology, extraction methods and study of some important bioactivities

S. B. Patel[1], U. A. Attar[1], D. M. Sakate[2] and S.G. Ghane[1]*

[1]Plant Physiology Laboratory, Department of Botany, Shivaji University, Kolhapur – 416 004, Maharashtra, India.

[2]Department of Statistics, Shivaji University, Kolhapur – 416 004, Maharashtra, India.

*Corresponding author: Tel.: +91 231 2609157; Fax: +91 231 269 2333.

Email address: sgg.botany@unishivaji.ac.in

Preliminary screening for the extraction of cucurbitacins

Solvent extraction is the most commonly used method for the extraction of metabolites. Generally, metabolites are present in the sample matrix either in simple or complex form. Therefore, the selection of solvent largely depends on nature and stability of metabolites and pre-requisite for the efficient extraction of desired metabolites[17,19]. Preliminary experiments were conducted in order to select an appropriate solvent for the extraction of CUI and CUB. Organic solvents, particularly acetone, chloroform, ethyl acetate, hexane, ethanol and methanol have been proved to be the most commonly used solvents in terpenoid extraction from the botanicals. In the present study, fruit powder extracted with chloroform revealed the highest recovery of CUI (0.310±0.05 mg/g DW) and CUB (0.128±0.03 mg/g DW) as compared to other solvents. In contrast, least CUI content (0.0271±0.02 mg/g DW) was noted in acetonitrile; hence, chloroform was used in further experiments (Supplementary Fig. S1a). To identify the factors required for the RSM, five independent variables such as SS ratio, maceration speed (rpm), mean particle size (PS), time and temperature were selected and the results are presented in Supplementary Fig. S1b,c. The SS ratio (1:20 to 1:60 g/mL) revealed an initial rise in CUI yield up to 0.317±0.02 and 0.086±0.02 mg/g DW but further increase in solvent volume resulted with decline in yield of both the cucurbitacins. In the present study, 1:30 g/mL SS ratio gave the highest CUI yield (0.317±0.02 mg/g DW), however, 1:20 g/mL SS ratio revealed highest CUB (0.0856±0.02 mg/g DW) content. The range of CUI was from 0.233±0.04 to 0.315±0.05 mg/g DW; wherein 80 rpm was noted to be the best for the recovery of CUI (0.315±0.04 mg/g DW). At the same time, CUB was maximum (0.0248±0.07 mg/g DW) at 40 rpm. Similarly, PS (150 to 850 µm) were tested and revealed that yield was inversely proportional to the particle size. The smallest particle size (150 µm) resulted into maximum CUI (0.906± 0.02 mg/g DW) and CUB (0.077±0.01 mg/g DW). Likewise, extraction time (30-120 min) was also tested and the highest CUI (2.3158±0.4377 mg/g DW)

and CUB (0.085±0.04 mg/g DW) content was noted at 60 and 30 min exposure, respectively. Further, increase in time exposure up to 2 h showed a significant decline in the yield. In addition, recovery of CUI was tested at varied temperature (30-60^{0}C), wherein, maximum recovery of CUI (1.507±0.03 mg/g DW) and CUB (1.061±0.03 mg/g DW) was noted at 50^{0}C (Supplementary Fig. S1b-c). Further, rise in temperature led to decrease in yield of cucurbitacins. On the basis of results from the study, three levels lowest, middle and largest (-1, 0 and +1, respectively) were decided and used in BBD.

Preliminary screening and selection of appropriate extraction parameters

Total seven different solvents, viz. acetone, ethanol, methanol, chloroform, acetonitrile, ethyl acetate and hexane were examined. Fruit powder (1 g) of 250 µm particle size and selected organic solvents were mixed separately in 250 mL conical flask with constant solute solvent ratio (1:30) and subjected to orbital shaker incubator at known conditions (extraction temperature 40°C, rotation per minute 120 rpm, time 60 min). Optimized extraction solvent 'chloroform' was used to finalize solute to solvent (SS) ratio including 1:20, 1:30, 1:40, 1:50 and 1:60 g/mL. The remaining extraction conditions were kept constant as above. The parameter rotation per minute (rpm) was optimized by selecting the best solvent (chloroform) and SS ratio (1:30 g/mL) whereas rpm levels were in between 80 to 200. Similarly, other parameters like particle size (150 to 450 µm), extraction time (30, 60, 90 and 120 min) and extraction temperature (30 to 60^{0}C) were optimized by keeping the other optimized parameters as constant. All the extracts were prepared as mentioned earlier and used for analysis.

17. Attar, U.A. & Ghane, S.G. Optimized extraction of anti-cancer compound - cucurbitacin I and LC–MS identification of major metabolites from wild Bottle gourd (*Lagenaria siceraria* (Molina) Standl.). *S. Afr. J. Botany*. **119**, 181-187 (2018).

19. Liyana-Pathirana, C. & Shahidi, F. Optimization of extraction of phenolic compounds from wheat using response surface methodology. *Food Chem.* **93**, 47–56 (2005).

Supplementary Fig. S1.

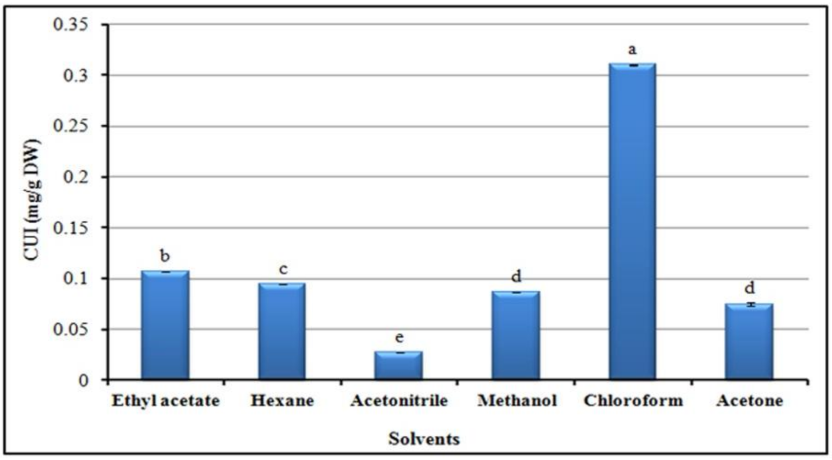

(a)

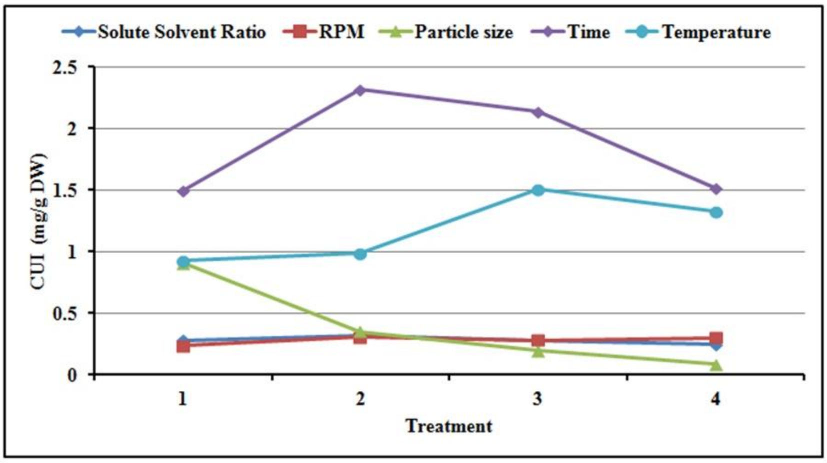

(b)

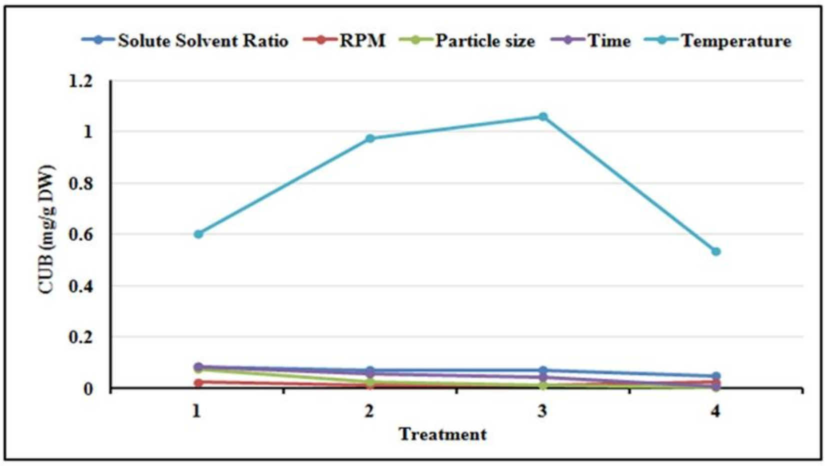

(c)

Supplementary Fig. S1. Preliminary screening experiments showing extraction of cucurbitacins using organic solvents (a), influence of solute to solvent ration, rpm, particle size, time and temperature on CUI (b), and CUB (c).

Supplementary Table A1
BBD with the independent variables and comparison of observed data with predicted for the responses (CUI, CUB and CUI+B)

Run[a]	Factors					Y1:CUI (mg/g DW)		Y2: CUB (mg/g DW)		Y3: CUI+B (mg/g DW)	
	A: SS ratio	B: rpm	C: PS	D: Time	E: Temp	Obs	Pred	Obs	Pred	Obs	Pred
1	20	80	450	60	50	0.3826	0.6680	0.2777	0.4313	0.6603	1.10
2	20	80	300	30	50	0.6194	0.5814	0.5696	0.4839	1.1890	1.07
3	30	80	300	90	60	0.6113	0.5670	0.4604	0.4132	1.0717	0.9801
4	30	40	300	90	50	0.7438	0.7009	0.6283	0.5133	1.3721	1.21
5	30	120	300	30	50	0.5211	0.7116	0.4734	0.6430	0.9945	1.35
6	30	80	300	60	50	0.7557	0.8370	0.5761	0.3058	1.3318	1.14
7	30	80	300	60	50	1.0117	0.8370	0.0178	0.3058	1.0295	1.14
8	30	40	300	30	50	0.5414	0.7610	0.4772	0.5147	1.0186	1.28
9	30	80	450	90	50	0.5164	0.7006	0.3505	0.3991	0.8669	1.10
10	30	80	150	90	50	1.1194	0.9922	0.8219	0.8148	1.9413	1.81
11	30	80	150	60	40	0.1775	0.6710	0.0175	0.5179	0.1950	1.19
12	20	80	300	60	40	0.5844	0.3218	0.5476	0.1487	1.1320	0.4704
13	30	120	150	60	50	1.2061	1.1700	1.1505	0.9540	2.3566	2.12
14	30	80	300	60	50	0.4680	0.8370	0.0508	0.3058	0.5188	1.14
15	40	80	300	90	50	0.5790	0.6335	0.4801	0.6501	1.0591	1.28
16	40	80	300	30	50	0.7673	0.8596	0.4953	0.6387	1.2626	1.50
17	30	40	300	60	40	0.7578	0.3849	0.5916	0.4300	1.3494	0.8150
18	30	40	450	60	50	0.0887	0.4156	0.0492	0.1981	0.1379	0.6137
19	40	40	300	60	50	0.7456	0.7520	0.5700	0.5657	1.3156	1.32

20	30	120	450	60	50	0.2862	0.3122	0.0705	0.1590	0.3567	0.4712
21	30	120	300	90	50	0.5135	0.4415	0.4453	0.4623	0.9588	0.9039
22	20	80	150	60	50	0.7939	0.7148	0.1230	0.3507	0.9169	1.07
23	30	80	450	60	60	0.2038	0.2407	0.1358	-0.0833	0.3396	-0.1241
24	20	80	300	60	60	0.5121	0.5201	0.4428	0.3017	0.9549	0.8218
25	30	40	300	60	60	1.3409	0.8602	0.2000	0.2740	1.5409	1.13
26	40	80	450	60	50	0.0927	0.0282	0.1431	-0.1093	0.2358	-0.0811
27	30	80	300	60	50	1.5004	0.8370	0.6529	0.3058	2.1533	1.14
28	30	80	300	30	60	0.8385	0.9421	0.5989	0.6759	1.4374	1.62
29	30	120	300	60	60	0.5681	0.4226	0.5723	0.5846	1.1404	1.01
30	40	80	150	60	50	2.2181	1.7900	1.5840	1.41	3.8021	3.19
31	40	120	300	60	50	0.5138	0.5149	0.5068	0.4926	1.0206	1.01
32	30	80	300	60	50	0.7761	0.8370	0.1055	0.3058	0.8816	1.14
33	30	80	450	60	40	1.0275	0.7780	0.1132	0.3469	1.1407	1.12
34	20	120	300	60	50	0.3020	0.3804	0.2003	0.3472	0.5023	0.7276
35	40	80	300	60	40	0.5109	0.5452	0.5039	0.4430	1.0148	0.9882
36	30	40	150	60	50	1.1106	1.3700	0.9737	0.8376	2.0843	2.21
37	30	80	300	60	50	0.5103	0.8370	0.4311	0.3058	0.9414	1.14
38	30	80	300	30	40	0.2686	0.5400	0.2707	0.3882	0.5393	0.9282
39	20	40	300	60	50	0.3683	0.4520	0.0398	0.1967	0.4081	0.6487
40	30	80	150	30	50	2.3450	1.7700	1.4648	1.21	3.8098	2.98
41	40	80	300	60	60	0.4261	0.7311	0.3249	0.5218	0.7510	1.25
42	30	80	150	60	60	1.3754	1.8700	1.1326	1.18	2.5080	3.05
43	30	120	300	60	40	0.5513	0.5137	0.4199	0.1966	0.9712	0.7103
44	30	80	450	30	50	0.5174	0.2534	0.3902	0.1885	0.9076	0.4420
45	20	80	300	90	50	0.5530	0.4773	0.3496	0.2905	0.9026	0.7677
46	30	80	300	90	40	0.4615	0.5849	0.4757	0.4690	0.9372	1.05

[a]Box-Behnken design constructed using 5 independent variables with 3 levels.

Supplementary Table A2

In vitro anticancer activity of *D. palmatus* fruit against human cancer cell lines.

	MCF-7			HT-29		
	GI_{50} µg/mL	TGI µg/mL	LC_{50} µg/mL	GI_{50} µg/mL	TGI µg/mL	LC_{50} µg/mL
Standard adriamycin	<10	104.27	385.17	<10	0.880	63.20
Diplocyclos fruit	<10	5.14	44.27	<10	68.31	46.88

TGI (µg/mL) (Concentration of drug causing total inhibition of cell growth), LC_{50} (µg/mL) (Concentration of drug causing 50% cell death), GI_{50} (µg/mL) (Concentration of drug cause 50% of maximal inhibition of cell proliferation)

Supplementary Fig. S2.

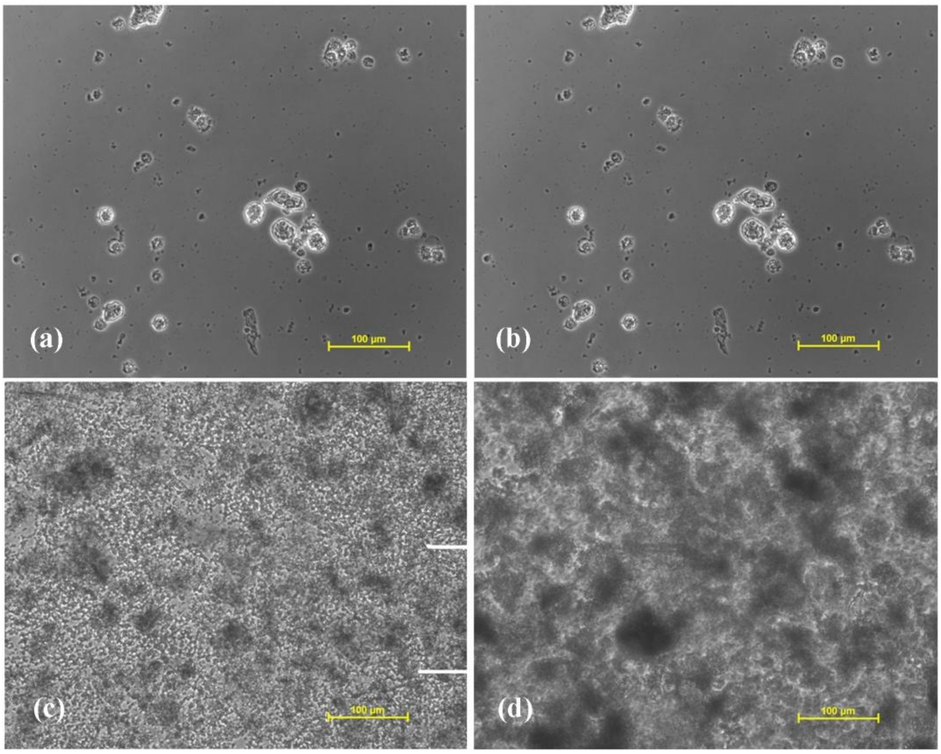

Supplementary Fig. S2. *In vitro* anticancer activity in *D. palmatus* fruit extract; positive control of HT-29 (a) and MCF-7 (b) and cell line treated with fruit extract HT-29 (c) and MCF-7 (d).

Supplementary Table A3

The compounds detected in fruit extract of *D. palmatus* by HPLC–ESI-Q-TOF-MS.

Sr. No	Compound	T_R	Molecular formula	Mass	M/Z	Category
1.	Caffeic acid 3-glucoside	0.65	$C_{15} H_{18}O_9$	342.095	365.08	Phenolic
2.	Digoxigenin monodigitoxoside	0.67	$C_{29} H_{44}O_8$	520.30	521.31	Steroid
3.	Isovaleric acid	2.57	$C_5 H_{10}O_2$	125.05	102.06	Fatty acid
4.	Digitalose	5.61	$C_7 H_{14}O_5$	178.08	179.09	Cardiac glycosides
5.	Luteolin 7-(2"-p-coumaroylglucoside)	6.62	$C_{30} H_{26}O_{13}$	594.134	595.14	Flavonoids
6.	Deserpidine	15.04	$C_{32} H_{38}N_2O_8$	578.26	579.26	Alkaloids
7.	Minabeolide-8	15.54	$C_{29} H_{42}O_5$	488.33	470.30	Sterols
8.	Beta-Hederin	15.60	$C_{41} H_{66}O_{11}$	752.491	734.45	Saponins
9.	Cucurbitacin I	15.90	$C_{30}H_{42}O_7$	514.288	497.285	Triterpene
10.	Cucurbitacin B	15.99	$C_{30}H_{46}O_8$	558.712	540.696	Triterpene
11.	Bufadienolide	16.13	$C_{24} H_{34}O_2$	377.24	354.25	Cardiac glycosides
